# Nurses’ beliefs about back pain, their coping strategies and participant activation for self-management

**DOI:** 10.4102/sajp.v78i1.1622

**Published:** 2022-10-20

**Authors:** Loveness A. Nkhata, Yolandi Brink, Dawn Ernstzen, Diribsa Tsegaye, Quinnette Louw

**Affiliations:** 1 Department of Physiotherapy, Faculty of Medicine and Health Sciences, Stellenbosch University, Stellenbosch, South Africa; 2 Department of Physiotherapy, Faculty of Health Sciences, University of Zambia, Lusaka, Zambia; 3 Department of Biostatistics, Faculty of Medicine and Health Sciences, Stellenbosch University, Stellenbosch, South Africa; 4 Department of Biostatistics, Faculty of Health Sciences, Wolkite University, Wolkite, Ethiopia

**Keywords:** nurses, back pain, beliefs, coping strategies, participant activation, self-management

## Abstract

**Background:**

Back pain affects nurses’ physical, social and emotional well-being, as they encounter difficulties in executing their social and occupational duties.

**Objectives:**

Our study investigated the impact of a cross-cultural back pain campaign on nurses’ beliefs about back pain; activating the participants to self-manage; coping strategies; sick leave claimed; and frequency of doctor visits.

**Method:**

A single sample pre- and post-test design was used. The intervention was a 12-week educational campaign based on evidence-based back pain messages. Primary outcomes were measured by their beliefs about back pain and their activation to self-manage. Analyses were conducted using SPSS version 27.0 software, and significant differences from before and after the campaign were analysed using the Chi-square test at a 0.05 significance level.

**Results:**

There were no significant differences in the age, gender and work hours of the nurses who participated before and after the campaign, except for their professional work settings (< 0.05). All secondary outcomes improved significantly after the campaign, and outcomes on beliefs about back pain showed significantly positive changes in six of the 14 items, while all questions pertaining to patient activation improved significantly.

**Conclusion:**

The 12-week back pain campaign, based on contextualised, evidence-based back pain messages for Zambian nurses, motivated the participants to self-manage their back pain. However, not all beliefs about back pain changed positively after the campaign.

**Clinical implications:**

The findings of this back pain education campaign show promise as a strategy to improve knowledge, behaviours and beliefs about back pain in African settings.

## Introduction

Back pain is a leading cause of morbidity and negatively affects physical functioning and work productivity (Abedini et al. 2014; Alnaami et al. 2019). Back pain refers to discomfort occurring anywhere in the thoracic and lumbar regions, with or without leg pain (Gim 2017). The prevalence of back pain among nurses ranges from 55% to 84%, globally (Freimann, Merisalu & Pääsuke 2015; Yan et al. 2004). In Zambia, the prevalence of back pain is about 65% (Nkhata et al. 2015). The high incidence of back pain among nurses leads to increased pressure on the national healthcare system and puts further strain on the nursing workforce (Alnaami et al. 2019; Gim 2017; Richardson et al. 2019). Back pain commonly restricts nurses’ ability to perform their social and occupational activities (Richardson et al. 2019; Yan et al. 2004). Handling patients; high caseloads; long working hours; poor ergonomics; workforce shortages; and a lack of equipment can increase the risk of back pain among nurses (Nkhata et al. 2016; Nkhata et al. 2020; Samaei et al. 2017; Tosunoz & Oztunc 2017). The lack of workplace support systems for nurses with back pain, as well as job dissatisfaction, contribute to reduced quality of life and physical dysfunction (Dlungwane, Voce & Knight 2018). In addition, socio-economic and other factors such as beliefs about management may exacerbate the nurses’ experience of back pain (Dressner & Kissinger 2018; Nkhata et al. 2021; Samaei et al. 2017).

Nurses with back pain often depend on non-evidence-based, passive management because of their limited knowledge about evidence-based back pain strategies, and they may fear that movement may exacerbate the symptoms (Budhrani-Shani et al. 2016; Nkhata et al. 2020). Passive methods, such as pain medication, have a limited effect on back pain and over-reliance can lead to negative effects, such as longer pain episodes, dependence and other side effects of medications (Traeger et al. 2019). Therefore, the use of passive strategies is not supported in evidence-based guidelines for back pain (Budhrani-Shani et al. 2016; Kusma et al. 2019). Hence, there are consistent recommendations in evidence-based guidelines for nonpharmacological approaches that include the active involvement of the individual, such as self-management to maintain or improve physical functioning and well-being (Ahmed et al. 2018; Hartvigsen, Natvig & Ferreira 2018).

Self-management is recommended for persistent health conditions, including back pain (Crowe et al. 2010; Dickson & McDonough 2018). Self-management refers to the efforts that individuals make to live with a chronic condition (Patel et al. 2019; Taylor et al. 2016). Education and behaviour modification are used to motivate people to actively participate in managing their health and lessening the condition’s negative effects on their physical functioning and well-being (Patel et al. 2019; Taylor et al. 2016). Individuals are provided with the tools they need to increase their knowledge and understanding of their health needs and take active control of their health (McCabe et al. 2018). Effective self-management of back pain requires people with the appropriate level of knowledge, skills and conviction to actively participate in decision-making and health management (Ahn et al. 2015; Green & Hibbard 2012; McCabe et al. 2018).

Participant activation is described as the desire and ability to self-sufficiently care for individual health needs and is related to self-management behaviours and health outcomes (Green & Hibbard 2012; Nkhata et al. 2021). People with higher levels of activation are more inclined to engage in self-management, because they have the confidence and knowledge to preserve or enhance their health in a responsible manner (Ahn et al. 2015). Hence, participant activation is a key strategy for achieving back care goals (Ahn et al. 2015; Green & Hibbard 2012). The implementation of self-management activities by affected persons may also be affected by perceptions about back pain (Grøn et al. 2019). Negative beliefs may emanate from poor health outcomes after seeking medical care, leading to increased pain and disability (Ahmed et al. 2018). Beliefs can be modified and are crucial in managing back pain (Carneiro, Bunzli & O’Sullivan 2020). Media campaigns are useful in improving coping and self-management in individuals with back pain by enhancing knowledge and perceptions of back pain (Traeger et al. 2019).

Back pain media campaigns comprise activities that increase awareness of the benefits of self-management through messages that encourage coping strategies, such as positive thinking, and dissuade the population from excessive use of pain relief medication and physical inactivity (Traeger et al. 2019). It has been reported that these campaigns have resulted in improved perceptions about health and positive healthcare-seeking behaviours, such as fewer sick leave days and doctor visits by participants, most of whom remain active after the campaigns, despite their back pain (Nkhata et al. 2019). Obtaining information on perceptions about back pain, coping strategies, participant activation, frequency of sick leave and doctor visits is important for decision-making and planning tailored healthcare activities (McCabe et al. 2018). Furthermore, the information is vital in developing applied and participant-centred back pain interventions relevant to the nursing profession. Our study aimed to investigate the impact of a 12-week back pain campaign targeting back pain beliefs, participant activation for self-management, coping strategies, sick leave claimed and frequency of doctor visits.

## Methodology

### Study design

A single-sample pre- and post-test design was used to conduct our study. This design is used when it is not ethically feasible to conduct a randomised control trial (RCT), or when there are logistical constraints (Harris et al. 2006). This design was chosen to avoid cross-contamination between hospitals because of the nature of the intervention, as the participating hospitals were located relatively close to each other. In addition, other hospitals within this region were not comparable, because of differences in the type and level of care and the intensity of services and resources, such as staff capacity, when compared to the participating hospitals. Besides, using a control group from another region may have introduced confounding factors that would be difficult to control for during analysis. The choice of design was based on methods used in similar campaigns (Buchbinder et al. 2008; Hoy et al. 2010; Waddell et al. 2007; Werner et al., 2007). The design was useful in generating general trends and reducing the time and resources needed for experimentation. Furthermore, because the design employed pre- and post-intervention measurements, it helped to highlight the intervention’s impact on, and benefits for, the target population (Harris et al. 2006; White & Sabarwal 2014).

### Setting for the study

Our study was carried out at four level-one public health facilities in Lusaka, Zambia. The facilities are resource-constrained settings that provide in- and outpatient health services, including public health programmes; maternal and child health activities; interventions for common conditions such as malaria and tuberculosis, human immunodeficiency virus (HIV) and acquired immunodeficiency syndrome (AIDS) programmes (LDHO 2017). The facilities were purposefully chosen as study sites because they were similar in operational systems, easily accessible and employed a substantial number of nurses.

### Sampling method and sample size

Our study population consisted of all nurses employed at the four health facilities, regardless of their back pain status at the time of our study. There were approximately 460 serving nurses in total at these facilities (LDHO 2018). It had been anticipated that most nurses would have experienced back pain at some time during their work life (Richardson et al. 2019). Therefore, the primary outcomes were appropriate for all nurses, regardless of their pain status during the period our study was conducted. A sample size of 322 was determined using McNemar’s test for paired proportions at 80% power and 5% significance level to determine a difference of 0.10 (Dhand & Khatar 2014). The principal nursing officers’ registers assisted in identifying and recruiting registered nurses and nurse managers working in the different hospital departments and who were available to participate in our study.

### Intervention description

The intervention was a 12-week educational campaign for nurses, based on evidence-based back pain messages, which were prioritised and contextualised in a preliminary study (Nkhata 2021). The campaign was aimed at altering unhelpful and incorrect perceptions of back pain, promoting health, increasing knowledge about back pain and enhancing the participants’ self-management.

#### Campaign kick-off session

**FIGURE 1:**
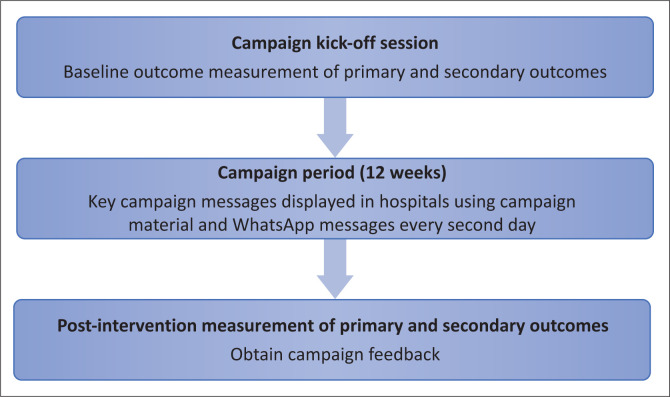
Campaign and outcome measurement process.

The first author arranged a campaign kick-off session. The same session was repeated a few times, as only 30 nurses could be accommodated in each group. During the kick-off session, vital information about the campaign (aim, roll-out and intended outcomes) was relayed to nurses in the form of a drama, so that nurses found the session entertaining (and this also served as an incentive for the nurses at the four participating hospitals to attend the kick-off session). The campaign procedures are shown in Figure 1.

#### Campaign messages

The campaign content included evidence-based messages that had been used in similar published campaigns. A systematic review of similar campaigns (Nkhata et al. 2019) was conducted and the evidence-based messages for back pain were extracted. The evidence-based messages were then cross-culturally validated and prioritised. A cross-cultural validation approach, using the Herdman et al. (1997) framework, was used to obtain insight, understanding of stakeholders’ perspectives and experiences and opinions of the synthesised back pain campaign messages on self-management and the extent to which they were applicable to the nurses in Zambia (Nkhata 2021). A total of 32 nurses participated in the cross-cultural adaptation of the messages. The messages selected by the nurses for this campaign included:

- Avoid taking unnecessary pain killers when you have back pain.
- There is a lot you can do to help yourself.
- Back pain is a personal responsibility, and it is up to you to look after your back.
- Back pain is rarely caused by a dangerous illness.
- The key to feeling better sooner is to stay active.
- Surgery is not the answer for back pain.

The nurses who participated in the cross-cultural validation also provided preferences on how and where the low back pain messages should be displayed. They suggested posters, blood pressure (BP) machine stickers, door stickers, branded pens and branded mugs.

#### Campaign communication material and messaging

**FIGURE 2:**
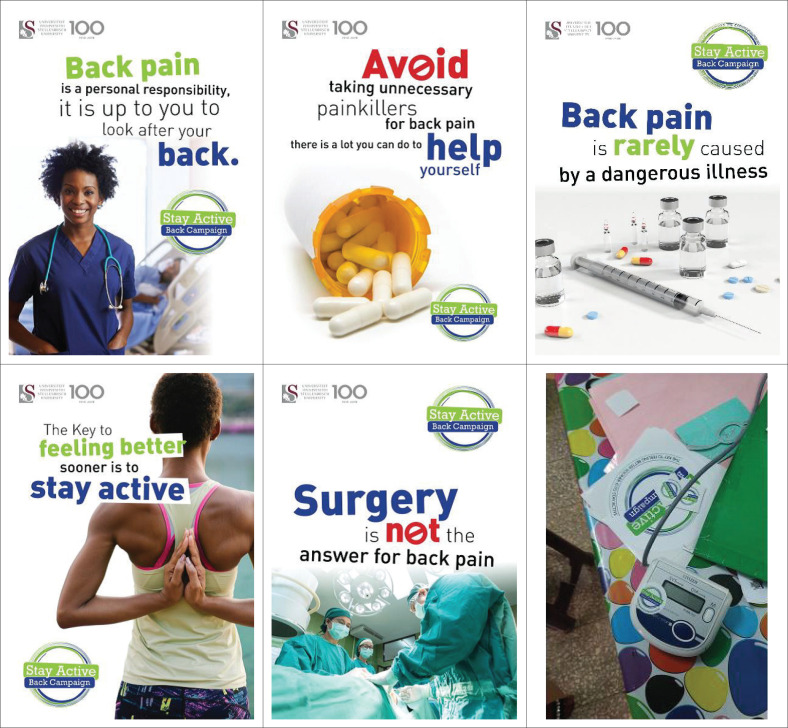
Samples of campaign materials used in delivery of back pain messages.

A graphic designer assisted with the design of the campaign material. The intervention period was 12 weeks, and the custom-designed campaign materials were used throughout the 12-week campaign. Figure 2 shows samples of the materials used in delivering the back pain messages. The campaign messages were displayed on posters, mugs, pens and BP machines, and stickers were placed on doors to optimise visibility to nurses at the selected hospitals.

The first author also used WhatsApp messaging to convey the same messages via videos every second day to the nurses at the participating hospitals.

### Outcomes and data collection

#### Demographic information

Demographic information collected included age, gender, level of education, work setting and work hours, using a data capture form. The participating nurses completed this form at baseline, and after the campaign this was done by a research assistant.

#### Primary outcomes

Primary outcome measurements obtained in our study included:

- beliefs about back pain
- participant activation for self-management.

To assess beliefs about back pain, a similar campaign reported and published in the United Kingdom (UK) used a set of self-constructed questions on beliefs about back pain and professional advice received (aligned with the key campaign messages: beliefs about back pain) to assess the effect of the campaign (Waddell et al. 2007). A Canadian campaign included the same set of back belief questions as the UK campaign and the Back Beliefs Questionnaire (BBQ). None of the published campaigns used patient activation as an outcome measurement.

To assess the effect of the campaign among Zambian nurses, a pragmatic approach was adopted that would encourage participation and overcome time constraints in completing the outcome scales. A questionnaire was constructed consisting of information available in the public domain. The constructed questionnaire consisted of items from the BBQ, as well as freely available public-domain items from the patient activation measurement (PAM) to provide an indication of back beliefs and patient activation before and after the campaign. A similar descriptive approach was also adopted to ascertain the proportion of positive and negative responses before and after the campaign, as in other published campaigns (Waddell et al. 2007). Since Zambian nurses prioritised the selected key campaign messages, the authors were interested in describing the outcome of the campaign on each of the items selected for inclusion in the questionnaire. Although all the original BBQ items were included, binary responses were chosen to facilitate time-efficient and easier administration, compilation, score calculation and data processing (Grassi et al. 2007). A similar approach has been tested and suggested for other health-related outcomes, such as the SF 36 (Grassi et al. 2007). A five-point Likert scale was initially adopted by the developers of the BBQ to reduce confusion (and time taken to complete) with the scoring of other tools, against which the BBQ was tested (Symonds et al. 1996). In addition, the responsiveness of the BBQ was only established in one study, and the minimum important change was not established (Ferreira & Kamper, 2020) for meaningful interpretation of the effect of the interventions. There was no indication in any literature about using public domain information in its items or how they were scored. This diminished the validity of the instrument in this context (Beidas et al. 2015). For these reasons, a descriptive approach has been adopted for collecting and describing the outcomes.

To assess intent (understanding) of the questionnaire (items on back beliefs and patient activation), it was piloted among 20 conveniently selected nurses at one of the nonparticipating public health facilities. English is Zambia’s official language; therefore, no translation was required because all participants had tertiary qualifications. The results of our pilot study indicated that participants had the ability to comprehend the instructions and understand the questionnaire items, the sequence of questions and the flow of statements. The participants suggested minor changes to the wording, such as: ‘one must rest when one has back pain’ from ‘back pain needs to be rested’. In addition, the phrase ‘back trouble’ was changed to ‘back pain’ for the back beliefs questions. The word patient was changed to ‘participant’ and ‘health condition’ was changed to ‘back pain’ for the patient activation questions. The participants also agreed with the simplified scoring of the questionnaire items, in light of their work commitments and available time to participate in our study.

#### Secondary outcomes

The secondary outcomes were self-reported using a custom-designed data capture form. The secondary outcomes included:

1. duration of back pain
2. number of participants who claimed sick leave days for back pain (past 2 months)
3. number of participants who claimed sick leave days in general (past 2 months)
4. number of doctor visits for back pain (past 2 months)
5. coping strategies: percentage of participants who reported using suggested coping strategies (medication, bed rest and exercises) for back pain.

#### Measurement time-frames of primary and secondary outcomes

Primary and secondary outcomes were measured at baseline (before) and again at exit after the 12-week campaign ended (week 13 since the campaign had commenced) by a research assistant.

#### Campaign feedback

Feedback from nurses who participated in the campaign was obtained. The short feedback questionnaire included three questions that were collected at the time when the research assistant (at the hospitals) collected the follow-up outcome measurement. The three questions were:

- Were you happy with the back campaign for nurses in Lusaka, Zambia?
- Do you think the campaign had an impact on your back care goals?
- Would you recommend this campaign to other facilities?

### Data analysis

Demographic data were descriptively analysed using SPSS version 27, and significant differences in demographics before and after the campaign were analysed using Chi-square analysis. The significance level was set at 0.05 for all analyses.

We used a descriptive analytical approach and dichotomous responses were analysed for all items of the back beliefs and patient activation questionnaire. This approach also allows comparison of selected items with published campaigns (Gross et al. 2007; Werner et al. 2007). To assess for differences in the primary and secondary outcomes before and after the campaign, Chi-square analysis was used (and 95% confidence intervals for differences in the proportion of correct responses before and after the campaign). The feedback on the campaign was presented descriptively using percentages of total responses.

### Ethical considerations

Ethical approval and clearance for our study was obtained for the project entitled ‘The effectiveness of a contextualised back pain campaign for nurses in Lusaka, Zambia’, from Stellenbosch University Health Research Ethics Committee (reference number: S18/06/125s; project ID 7431); the University of Zambia Health Sciences Research Ethics Committee (protocol ID 20181016002), the National Health Research Authority, the Lusaka District Health Office and the participating health centres. All participants provided written, informed consent before participating in our study.

## Results

### Participants’ demographic descriptions

Table 1 shows that there were no significant differences in the age, gender and work hours of the nurses who participated, before and after the campaign. Statistically significant differences were found in professional work settings.

**TABLE 1:** Participants’ demographic descriptions.

Participants’ demographic descriptions	Before (*N* = 327)		After (*N* = 325)		*p*
	*N*	%	*N*	%	
**Age in years**					0.79
18–25	117	35.70	125	38.40	
26–35	126	38.50	127	39.00	
36–45	75	22.94	65	20.00	
46–60	9	2.50	8	2.40	
**Gender**					0.95
Male	103	31.50	102	31.20	
Female	224	68.50	224	68.70	
**Work setting**					< 0.05
Medical	118	36.80	53	16.50	
Paediatric health	55	17.90	28	8.70	
Theatre	14	4.30	41	12.70	
Surgical	6	1.80	32	9.90	
Maternity	49	15.30	48	14.90	
OPD	62	19.30	113	35.20	
Others	16	5.00	6	1.80	

Note: Work hours before (*N* = 327) - mean = 36.31; SD = 11.15; median = 40; IQR = 6. Work hours after (*N* = 325) - mean = 37.59; SD = 4.38; median = 40; IQR = 4. *p* = 0.07.

OPD, outpatient department; IQR, interquartile range; SD, standard deviation.

### Primary outcomes

The questionnaire on back beliefs showed that there were significant positive changes in 6 of the 14 back belief items. However, there were significantly worse scores in four of the back belief items (Table 2).

**TABLE 2:** Questions on back beliefs.

Back beliefs questionnaire items	Correct before campaign		Correct after campaign		*p*	95% CI of difference	Total responses (included disagreed and no response)
	*N*	%	*N*	%			
Q1 There is no real treatment for back pain	107	36.40	201	68.80	< 0.01*	24 – 39	292
Q2 Back pain will eventually stop you from working	118	37.80	97	31.00	0.29	−1 – 13	312
Q3 Back pain means periods of pain for the rest of one’s life	221	70.10	175	55.50	< 0.01*†	7 – 22	315
Q4 Doctors cannot do anything about back pain	36	11.30	79	25.00	< 0.01*	2 – 8	316
Q5 A bad back should be exercised	242	79.20	205	66.30	0.03*	6 – 19	309
Q6 Back pain makes everything worse in life	117	38.60	94	31.00	0.29	−0.01 – 14	303
Q7 Surgery is the most effective way to treat back pain	264	85.90	290	94.40	0.05*	3 – 13	307
Q8 Back pain may mean you will end up in a wheelchair	230	75.10	225	73.50	0.87*	−5 – 7	306
Q9 Alternative treatments are the answer to back pain	196	63.20	124	40.00	< 0.01*	9 – 35	310
Q10 Back pain means lengthy periods of time off work	168	54.50	133	43.10	0.08*	4 – 19	308
Q11 Medication is the only way to relieve back pain	181	59.10	286	93.40	< 0.01*	20 – 27	306
Q12 Once you have had back pain, there is always a weakness	137	45.80	190	63.50	< 0.01*	10 – 25	299
Q13 Back pain must be rested	27	8.70	95	30.90	< 0.01*	49 – 62	307
Q14 Later in life, back pain gets progressively worse	74	23.60	111	35.40	0.68	−19 – 4	313

* , statistically significant at level 0.05.

† , indicate worse responses after the campaign.

All questions pertaining to patient activation improved significantly after the campaign (Table 3).

**TABLE 3:** Items on participant activation.

Items on participant activation	Agreed before campaign		Agreed after campaign		*p*	95% CI of difference	Total responses (Included disagreed or no response)
	*n*	%	*n*	%			
1. When all is said and done, I am the person who is responsible for managing my back	187	60.70	273	88.60	< 0.01	16 – 39	308
2. Taking an active role in my back care is the most important factor in determining my health and ability to function	182	57.90	279	88.80	< 0.01	19 – 42	314
3. I am confident that I can take actions that will help me prevent or minimise some symptoms or problems associated with back pain	185	59.60	281	90.60	< 0.01	19 – 43	310
4. I am confident that I can tell when I need to get medical care and when I can handle back pain myself	160	53.60	268	89.90	< 0.01	24 – 47	298
5. I am confident that I can follow through on medical advice and treatment I need to do at home for back pain	193	63.20	239	78.30	0.02	2 – 27	305
6. I understand the nature and causes of back pain	136	45.90	266	89.80	< 0.01	32 – 55	296
7. I know the different treatment options available for back pain	116	40.90	243	85.80	< 0.01	33 – 56	283
8. I have been able to maintain the lifestyle changes for back pain that I have made	127	43.40	247	84.50	0.01	35 – 58	292
9. I know how to prevent further problems with back pain	120	42.10	254	89.10	< 0.01	40 – 53	285
10. I am confident I can figure out solutions when problems with back pain arise	132	46.30	263	92.20	< 0.01	34 – 57	285
11. I am confident that I can maintain lifestyle changes like diet and exercise, even during times of back pain	132	44.70	259	87.70	0.01	31 – 54	295

### Secondary outcomes

All secondary outcomes (Table 4) pertaining to the duration of back pain, sick leave and doctor visits for back pain improved significantly (except for the number of participants who claimed sick leave days). Participants used significantly less pain medication, and although there were positive trends in the use of ‘bed rest’ and ‘exercises’, these outcomes were not significantly different after the campaign (Table 5).

**TABLE 4:** Participants’ back pain history, sick leave days and frequency of doctor visits (during intervention period.

Participants’ back pain history	Before		After		*p*
	*n*	%	*n*	%	
**Typical duration of back pain**					< 0.01
1–3 days	109	53.70	124	91.80	
4–7 days	70	34.15	11	8.00	
More 2 weeks	26	12.68	1	0.74	
**Participants who claimed sick leave because of back pain**					< 0.01
Total participants	84	34.00	59	21.50	
**Participants who claimed sick leave days**					0.26
1–3 days	124	91.80	75	23.70	
4–7 days	12	8.00	5	3.70	
More than a week	1	0.74	2	2.50	

Note: Mean number of doctor visits for back pain = 1.71, standard deviation 0.96. Mean number of doctor visits for back pain after = 0.46, standard deviation 0.67. *p* = < 0.001.

**TABLE 5:** Participants’ coping strategies.

Coping strategies for back pain	Before		After		*p*	95% CI
	*n*	%	*n*	%		
Number of participants who used pain medication	267	81.60	126	38.70	0.01*	30 – 55
Number of participants who used rest for back pain	117	35.90	85	26.20	0.16	−3 – 21
Number of participants who exercised or used physiotherapy	87	26.50	111	34.20	0.21	−20 – 4

* , statistically significant at level 0.05.

### Participants’ feedback on campaign

Feedback on the campaign highlighted (Table 6) that almost all participants were happy with the back pain campaign for nurses in Zambia. In addition, most participants reported that the campaign had influenced their back care goals and recommended that the campaign activities should be conveyed to other health facilities.

**TABLE 6:** Campaign feedback.

Feedback question	Yes		No		Total
	*n*	%	*n*	%	
Were you happy with the back campaign for nurses in Lusaka, Zambia?	312	96.30	12	3.70	324
Do you think the campaign had an impact on your back care goals?	313	95.90	11	4.10	324
Would you recommend this campaign to other facilities?	304	99.30	2	0.65	306

## Discussion

Our article reports on the first back pain campaign conducted among nursing professionals in a low- and middle-income setting in Africa. We assessed the effects of the back pain campaign on beliefs, participant activation for self-management and healthcare utilisation for back pain among nurses in Lusaka, Zambia. We argued that beliefs are modifiable and that context-specific educational interventions can be an important strategy to change beliefs and healthcare behaviours regarding back pain (Carneiro et al. 2020; Grøn et al., 2019), as this changes awareness, reduces disability and enhances coping in individuals with back pain. The main findings are that the 12-week Zambian back pain campaign for nurses activated the participants to self-manage back pain. Although fewer than half of the back belief items indicated a positive change, these were congruent with the six campaign messages. The gains in knowledge, with consequent changes in belief and confidence in self-management, led to decreased healthcare utilisation for back pain.

There were positive changes in beliefs regarding the role of doctors, medication, surgery and the role of movement in back pain. However, participants had conflicting beliefs regarding the prognosis of back pain and the role of exercise and alternative treatment strategies for back pain. The findings may be explained in that the six main campaign messages focused on the role of medical management and rest versus movement, but they did not focus on messages about prognosis, exercise and alternative management approaches. Although the number of messages included in this campaign aligned with published campaigns, it is suggested that, in future, campaigns of this nature could consider alternating messages over a longer period to address all aspects of beliefs. In addition, the mixed findings regarding beliefs of the participants may be explained by context job-related factors (such as long hours of job-related physical activity) and nurses’ previous experiences with back pain (Nkhata 2021). Job-related factors include aspects such as workload, which is frequently impacted by work hours, human resources and task execution (Nkhata et al. 2020). Participants may have been accustomed to taking leave and resting when having back pain, which contradicts the notion of exercising when you have back pain. Hence, understanding nurses’ previous experiences with back pain and its management, as well as their lifestyle habits and training regarding back pain, may be key to the change in beliefs. Future campaigns should therefore design approaches that are based on evidence-based back pain practices and which better incorporate the context of the intended population.

A key outcome of back pain campaigns is a change in beliefs, which, it is envisaged, can assist in self-management behaviours. Self-management is an integral part of back pain management, as it promotes health and function (Burd & Hallsworth 2017). The ability to self-manage can be measured by a reduction in healthcare utilisation, including a decrease in the frequency of doctor visits and sick-leave days and coping strategies used for back pain (McBain, Shipley & Newman 2015). Our study’s results indicate a significant positive shift in self-management behaviours, reported as less sick leave claimed, fewer visits to the doctor, less use of medication for back pain and fewer sick days. Congruent with the findings of back beliefs regarding exercise for back pain (Table 2, Q5), the participants reported that their level of exercising did not change significantly. Thus, despite their beliefs regarding rest for back pain changing significantly (Table 2, Q13), this did not result in a change in coping strategy. Our study findings regarding healthcare utilisation are similar to those from studies conducted in Australia and Scotland (Buchbinder et al. 2008; Werner et al. 2007). The above findings regarding healthcare utilisation is important for low-resource settings like Zambia, where the healthcare budgets are often limited by fiscal constraints. The finding also means that more nurses stayed at work despite back pain, as they arguably felt equipped to self-manage their back pain. This finding is encouraging, since it implies less pressure on an already strained health system, with limited workforce capacity. An additional benefit includes reduced healthcare related to healthcare utilisation. From the feedback obtained, the back campaign conveyed to nurses in Lusaka, Zambia, shows promise in enhancing self-management activities and related practices within the nursing profession. The back pain campaign activated self-management initiatives in the participants, promising a cost-effective public health approach when implementing a contextualised back pain campaign in similar contexts.

When comparing the outcomes of our study with international studies that focused on media campaigns for back pain, the results are promising. The Canadian campaign (Gross et al. 2010) detected a positive trend on the BBQ, yet the findings were not statistically significant for the overall effect of the campaign. Additionally, Gross et al. (2010), found no meaningful changes in the BBQ’s total score on specific items for nursing students participating in the campaign. Buchbinder et al. (2001) found that there was a large, statistically significant, improvement in back pain beliefs on the BBQ in one state in Australia but no change in another state. The Australian back pain campaign successfully influenced population attitudes, beliefs, health risk behaviours and reduction in absence because of sickness in the one state (Buchbinder et al., 2001, 2008). A Scottish campaign (Waddell et al. 2007) resulted in a significant shift in public beliefs regarding staying active, with a concomitant reversal in beliefs about rest. Likewise, in Norway, Werner (2008) found small improvements in the population’s back beliefs, specifically regarding beliefs about the use of X-rays, and return to work. However, there was no significant shift in the overall population’s sickness behaviour and back beliefs. Werner (2008) found a small but significant shift towards more positive self-coping attitudes. The above outcomes indicate that, although the outcomes of back pain campaigns are variable, the campaigns are useful in influencing certain beliefs and attitudes to back care goals and in promoting healthy behaviours in different populations. The outcomes of the campaigns seem to be related to the focus of the key messages provided, the timeframe, the budget and the scope of the campaign (Buchbinder et al. 2008).

### Strengths and limitations

Our study was designed based on evidence from previous back campaign messages (Nkhata et al. 2019), as well as context-specific information. The feedback on the campaign was overwhelmingly positive. Stakeholder and potential end-user involvement in implementing the design strengthened the approach and led to innovative methods to distribute the campaign messages. Including nursing personnel in the designing of the campaign, cross-culturally validating the back pain messages for understanding and having face-to-face interactions with the participants may have influenced the positive feedback. While previous studies focussed on the outcomes of back pain beliefs, outcomes were also included regarding activation for self-management, as well as the strategies used to cope when having back pain. The above information may be useful in designing future back pain educational interventions. Campaign materials were repeatedly displayed through the intervention period. This is an important factor in the duration of the effect that motivated participants’ activation for self-management and must be considered in future campaigns.

The use of self-administered questionnaires could have caused response bias. Participants may have responded to some by providing socially desirable answers (since the items were linked to campaign messages). Participants may also have exaggerated or omitted their back pain experiences and potentially introduced recall and report biases. The questionnaire used in our study was constructed based on items of the BBQ and publicly available items from the PAM and was only assessed for intent, but reliability studies are recommended in future studies. During data analysis, the overall scores for the instruments were not used. However, it was beneficial to analyse the items on the BBQ separately, as this provided clearer indications of the effect of the specific campaign messages. It is also important to note that the campaign was limited to level-one hospitals in Lusaka, and information obtained was based on individual reports that may be specific to participants’ work contexts. Thus, recommendations from our study may only be applied to similar contexts, even though similar situations may occur in other settings. Nevertheless, our study provides additional insights into the development and appropriateness of research evidence in resource-constrained settings.

## Conclusion

The 12-week back pain campaign, based on contextualised, evidence-based back pain messages for Zambian nurses, activated the participants to self-manage their back pain. The campaign also resulted in significant changes in some back beliefs, although not all back beliefs changed positively after the campaign. The increase in knowledge about back pain, with consequent changes in beliefs and capacity to self-manage, led to decreased healthcare utilisation and use of pain medicine for back pain. Although trends in the enhanced use of coping strategies were positive, these changes were not significant. The findings of this back pain education campaign show promise as a strategy to improve knowledge, behaviours and beliefs about back pain in African settings. Further research is required to provide guidance on the most relevant outcomes for the local context, duration and sustainability of the campaign outcomes, as well as the transferability of the campaign findings to the general Zambian population.
